# A Longitudinal Cohort Study of Risk Factors Associated with Small Ruminant Lentivirus Seropositivity in Intensively Reared Dairy Ewes in Greece

**DOI:** 10.3390/pathogens12101200

**Published:** 2023-09-27

**Authors:** Aphrodite I. Kalogianni, Ilias Bouzalas, Ioannis Bossis, Athanasios I. Gelasakis

**Affiliations:** 1 Department of Animal Science, School of Animal Biosciences, Agricultural University of Athens, 11855 Athens, Greece; gelasakis@aua.gr; 2 Hellenic Agricultural Organization-DEMETER, Veterinary Research Institute, Campus of Thermi, 57001 Thessaloniki, Greece; bouzalas@elgo.gr; 3 Department of Agricultural Sciences, School of Agriculture, Forestry and Natural Resources, Aristotle University of Thessaloniki, 54124 Thessaloniki, Greece; bossisi@agro.auth.gr

**Keywords:** small ruminant lentiviruses, sheep, risk factors, longitudinal seroepidemiological study, serological patterns, seroconversion, seroreversion

## Abstract

A two-year longitudinal cohort study was conducted on a total of 407 purebred Chios and Lacaune ewes from four intensive dairy sheep farms to assess potential risk factors for small ruminant lentiviruses (SRLVs) seropositivity. Ewes were serologically tested semiannually at pre-mating and pre-lambing, and their age, breed, and body condition score (BCS) were recorded. Εwes were categorized as constantly seronegative, constantly seropositive, seroconverted, seroreverted, or animals with an intermittent presence of antibodies. Mixed binary logistic regression models were used to estimate the adjusted relative risks of the studied risk factors for (i) the individual ewes’ seropositivity, (ii) the manifestation of specific serological patterns, and (iii) the occurrence of seroconversion and seroreversion incidents. Increased age was associated with seropositivity and constantly seropositive status (*p* < 0.001 in both cases). On the other hand, age was negatively associated with constantly seronegative pattern, seroconversion incident, and the intermittent presence of antibodies (*p* < 0.05 in all cases). Moreover, breed was recognized as a risk factor: Lacaune ewes demonstrated increased seropositivity, whereas Chios ewes were more likely to demonstrate an intermittent presence of antibodies (*p* < 0.01 in both cases). Seropositive status (*p* < 0.001), seropositivity in animals with an intermittent presence of antibodies (*p* = 0.001), and seroconversion incidents (*p* < 0.001) were significantly increased at pre-lambing compared to pre-mating. The risk factors recognized in our study contribute to a better understanding of SRLVs epidemiology and the evidence-based designation of SRLVs’ control programs in intensive dairy sheep farms in Greece.

## 1. Introduction

Small ruminant lentiviruses (SRLVs) belong to the *Retroviridae* family and infect both sheep and goats, causing the chronic diseases of maedi-visna (MV) and caprine arthritis encephalitis (CAE) [1]. Chronically infected animals may develop pneumonia, mastitis, arthritis, encephalitis, and progressive emaciation [2]. Although the severity of these clinical signs varies among infected animals, and in many cases the signs of the disease are not evident, SRLVs infections have been associated with reduced productivity, impaired health and welfare status, and increased replacement rate in infected small ruminant flocks [3,4,5,6,7,8,9,10,11,12].

As there is no treatment or vaccination against SRLVs infections, and considering the substantial economic losses, it is therefore essential that small ruminant farms undertake preventive measures and national surveillance programs [13,14,15,16]. The selection and implementation of effective preventive measures and successful control programs have been adversely impacted by the lack of specific epidemiological data for each small ruminant species according to their productive orientation, as well as for various geographical regions and farming systems. As a result, a global spreading of SRLVs infections has been reported, especially in countries with intensive sheep and goat farming.

The identification and quantification of potential risk factors can contribute to the development and implementation of cost-effective strategies to monitor, prevent, and mitigate SRLVs infections. Currently, a number of possible risk factors have been identified as being related to SRLVs seropositivity, both at the animal (e.g., age, breed, sex) and farm levels (e.g., intensive farming system, increased flock size, unfavourable housing conditions, breeding stocks trade, etc.) [4,15,17,18,19,20,21,22,23,24,25,26,27]. In all cases, however, the study design referred to cross-sectional epidemiological studies, including a wide range of farms, breeds, and animals reared under various farming systems. However, the primary issue of these epidemiological studies was the imperfect diagnostic performance of the applied serological diagnostic techniques, which might have resulted in the misclassification of animals based on a single observation of their serological status. In contrast, longitudinal cohort studies reduce the impact of diagnostic errors and enable the drawing of safer conclusions regarding the epidemiology and risk factors of chronic diseases [28,29].

In Greece, the dairy sheep industry is well-established and the largest contributor to the national livestock output. The rising demand for high-yielding breeding stocks has lately led to a remarkable increase in the importation of breeding stocks from European countries and/or from local intensive farms with unknown, or in some cases, high SRLVs prevalence rates. Despite this, the epidemiological data regarding SRLVs infections in the country are scarce [30,31], and there is limited information on the function of putative risk factors.

The gap of knowledge regarding risk factors and their association with the epidemiology of SRLVs infections in Greece motivated us to conduct a longitudinal seroepidemiological study in intensively reared dairy sheep. The objectives of this study were to prospectively study the potential risk factors associated with (i) the animal seropositivity, (ii) the manifestation of specific serological patterns, and (iii) the occurrence of seroconversion or seroreversion incidents.

## 2. Materials and Methods

### 2.1. Farms and Animal Population

The selection of the farms and animals has been already detailed by Kalogianni et al. [32]. In brief, a total of ten intensive dairy sheep farms with the two most common high-yielding dairy breeds in Greece, namely, Lacaune and Chios breed, were on-site surveyed to collect data regarding farms’ characteristics and management practices. Among them, a total of four representative intensive dairy sheep farms were enrolled in this study: two farms with purebred Chios (farms B and C), one farm with purebred Lacaune sheep (farm D), and one mixed farm with purebred Chios and Lacaune sheep (farm A). For the appropriate representation of all age classes in the study, the group of early lambed ewes in each studied farm was selected, leading to a total of 660 ewes (430 Chios and 230 Lacaune) from 6 months to 7 years old. The studied animals were separately penned within each farm during the whole period of the study.

### 2.2. Blood Sampling and Serological Analysis

Sampling, recording, and serological analysis with the ELISA test were performed semiannually for 2 consecutive years, 3–4 weeks pre-mating and 2 weeks pre-lambing, as previously described [32]. Blood samples were analysed for the detection of anti-SRLV antibodies with an indirect whole virus (OLV 130/91 strain/A genotype) commercial ELISA test (ELISA, CAEV/MVV Total Ab Test, IDEXX). The analytical sensitivity and specificity values of the ELISA test were 95.5% and 97.2%, respectively, when compared to a recombinant GAG (group-specific antigens)-GST (glutathione S-transferase) fusion protein expressed in *E. coli* ELISA [33]. Also, in each sampling, ewes were physically examined and the clinical signs associated with SRLV infection were recorded. Recordings included BCS (1–5: 1 = emaciated, 5 = obese, with 0.25 increments) [34], the occurrence of arthritis, respiratory disease (cough and abnormal respiratory sound), and mastitis. Ear tag, breed, and age were also recorded.

### 2.3. Statistical Analyses

For the statistical analyses, only the animals with at least four consecutive recordings were used (i.e., 407 animals: 234 Chios and 173 Lacaune ewes). Based on the evolution of their serological status during the 2 years of this study, ewes were classified into five serological patterns, namely, constantly seropositive (exclusively seropositive results during the study), constantly seronegative (exclusively seronegative results during the study), seroconverted (seronegative animals at the beginning of the study that converted to seropositive during the study), seroreverted (seropositive animals at the beginning of the study that reverted to seronegative during the study), and animals with an intermittent presence of antibodies (alternating seropositive and seronegative status due to fluctuating antibody titers, regardless of their serological status at the beginning of the study). Seroconversion/seroreversion incidents were defined as the time-points of seroconversion/seroreversion events, namely, the first sampling occasion that the animal was found to have seroconverted/seroreverted.

For descriptive statistics, animals were categorized in five age classes at the beginning of the study, namely, 1 (x ≤ 1 year-old), 2 (1 < x ≤ 2 years old), 3 (2 < x ≤ 3 years old), 4 (3 < x ≤ 4 years old), and 5 (x > 4 years old). Multivariable-adjusted relative risks (RR) for (i) seropositive status, (ii) the occurrence of various serological patterns, and (iii) seroconversion and seroreversion incidents were calculated using SPPS v.26 and mixed binary logistic regression models. Risk factors for serological patterns during the study were estimated using the breed (two levels, Chios and Lacaune) and the age of the animals at the beginning of the study (covariate) as fixed effects and the farm as a random effect. In the cases of the adjusted RRs for (i) seropositive status, (ii) seroconversion and seroreversion incidents, and (iii) seropositive status exclusively in animals with an intermittent presence of antibodies, repeated measures mixed binary logistic models were used. In the latter models, breed (two levels, Chios and Lacaune), sampling occasion (two levels, pre-mating and pre-lambing), and year of the study (three levels, first, second, third; in the third year only the sampling occasion at the pre-mating period was available) were used as fixed effects, while age and BCS were considered as covariates and farm and animal as random effects. Scaled identity was selected as the most appropriate covariance structure among variance component, first-order autoregressive, diagonal, compound symmetry, and unstructured, according to Akaike’s information criterion (AIC).

## 3. Results

### 3.1. Seroprevalence and Serological Patterns

Seroprevalence during the study ranged from 57.5% (first sampling occasion) to 75.4% (fourth sampling occasion) (Table 1).

**Figure 1:**
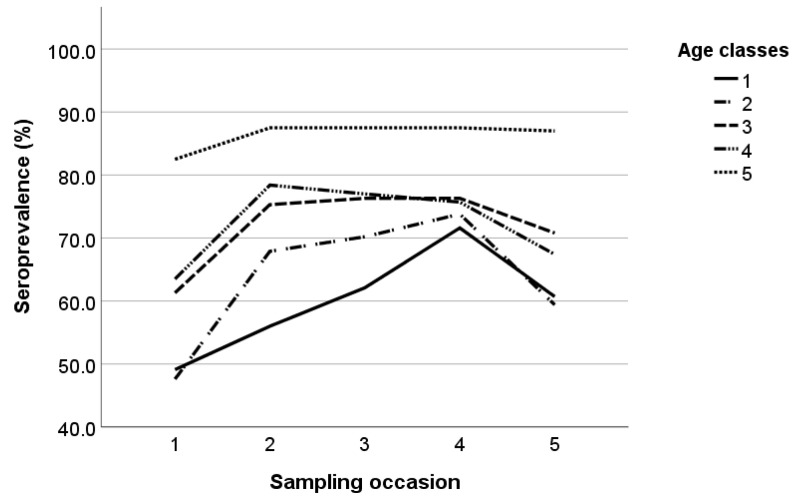
Small ruminant lentiviruses’ seroprevalence during the study per age class; the five age classes were 1 (x ≤ 1), 2 (1 < x ≤ 2), 3 (2 < x ≤ 3), 4 (3 < x ≤ 4), and 5 (x > 4).

Seroprevalence of SRLVs per age class is presented in Figure 1. During the study, seroprevalence increased in age classes 1, 2, and 3 until the fourth sampling occasion and then decreased in the last sampling. Although seroprevalence in age class 4 initially increased, it followed an earlier declining trend compared to the other three age classes during the study. On the other hand, seroprevalence in age class 5 remained almost constant during the study.

**Figure 2:**
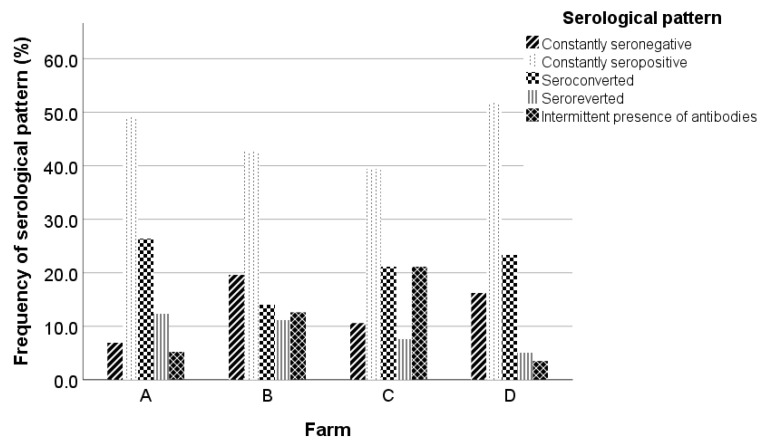
Frequency of serological patterns in the studied farms.

Constantly seronegative ewes were 15.2% (62/407), while constantly seropositive ewes were 46.2% (188/407). Ewes that either seroconverted or seroreverted once during the study were 20.1% (82/407) and 8.1% (33/407), respectively. Moreover, 10.3% (42/407) of the studied animals demonstrated an intermittent presence of antibodies during the study. Serological patterns per farm are presented in Figure 2. The percentages of constantly seronegative and seropositive animals varied from 7.0% in farm A to 19.6% in farm B, and from 39.4% in farm C to 51.8% in farm D, respectively. The lowest percentages of seroconverted and seroreverted animals were observed in farms B and D (14.0% and 5.0%, respectively), whereas the highest values of both serological patterns were observed in farm A (26.3% and 12.3%, respectively). The percentage of the intermittent presence of antibodies varied from 3.5% in farm D to 21.2% in farm C.

**Figure 3:**
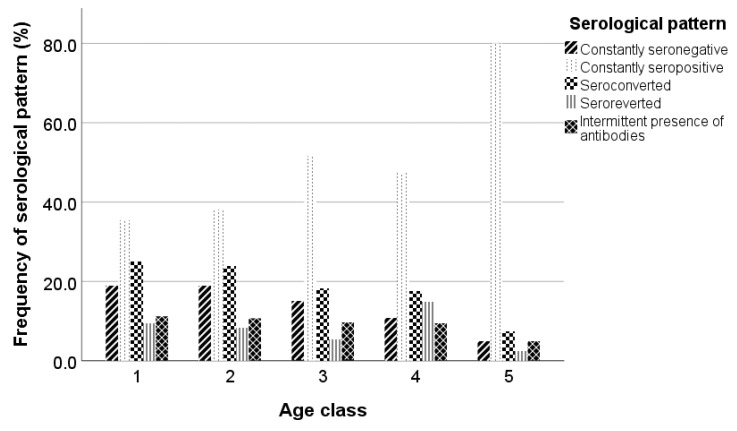
Frequency of serological patterns in each age class; the five age classes were 1 (x ≤ 1), 2 (1 < x ≤ 2), 3 (2 < x ≤ 3), 4 (3 < x ≤ 4), and 5 (x > 4).

The frequencies of serological patterns per age class are presented in Figure 3. Constantly seronegative and seroconverted animals reduced by age class, whereas constantly seropositive animals increased by age class until age class 3, decreased in age class 4, and increased again in age class 5. Also, age class 4 demonstrated a higher percentage of seroreverted animals, whereas animals with an intermittent presence of antibodies were similar among the age classes and reduced only in age class 5.

**Figure 4:**
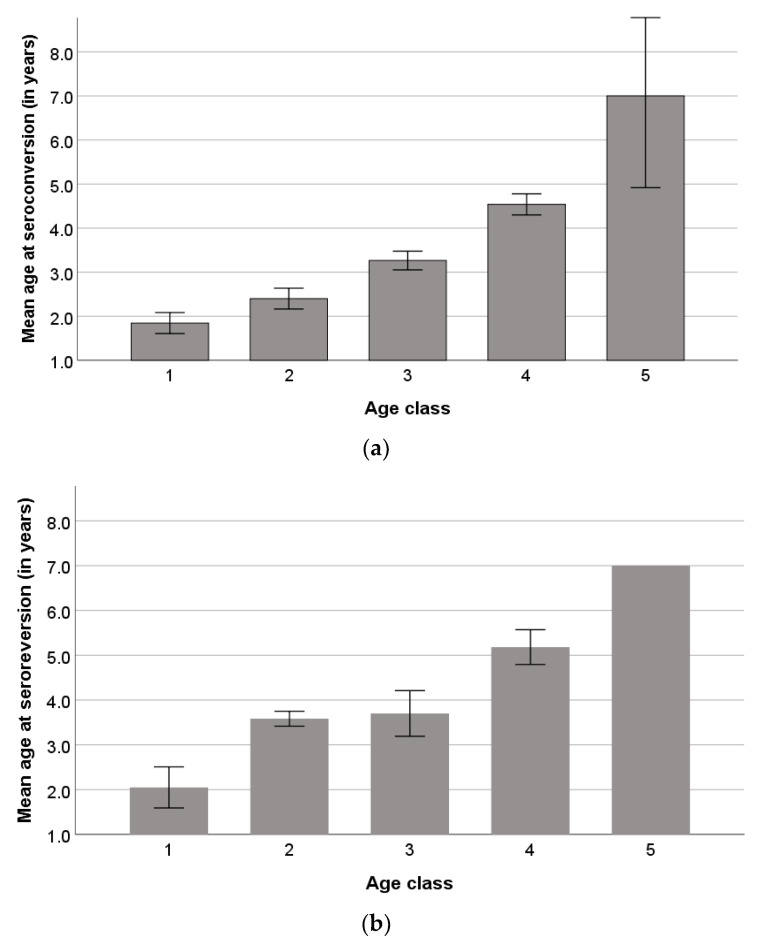
Mean ages (±S.E.) of animals at the seroconversion (**a**) and seroreversion (**b**) incident per age class. Age classes refer to the age of the studied animals at the beginning of the study; the five age classes were 1 (x ≤ 1), 2 (1 < x ≤ 2), 3 (2 < x ≤ 3), 4 (3 < x ≤ 4), and 5 (x > 4). The calculation of S.E. is not applicable in age class 5 of (**b**), as only one animal belonged to this age class.

The mean ages of animals at the seroconversion and seroreversion incidents were 2.9 ± 1.38 years and 3.8 ± 1.51 years, respectively. The mean ages of seroconverted and seroreverted animals at the seroconversion and seroconversion incidents per age class are presented in Figure 4a,b.

### 3.2. BCS and Health Recordings

**Figure 5:**
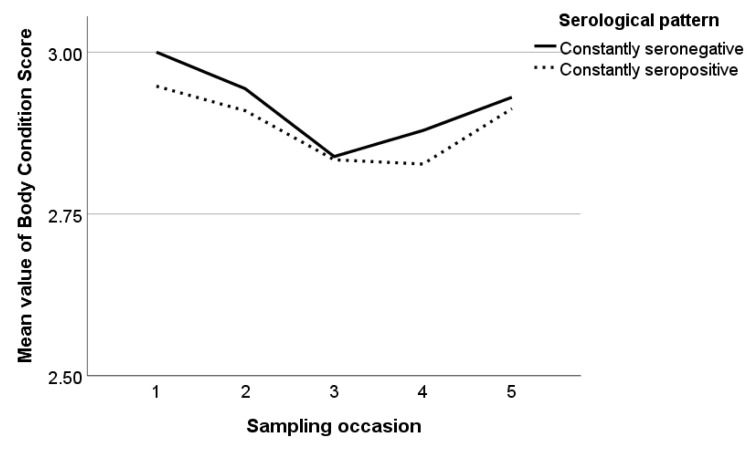
Mean values of body condition score during the study in constantly seronegative and constantly seropositive animals.

**Figure 6:**
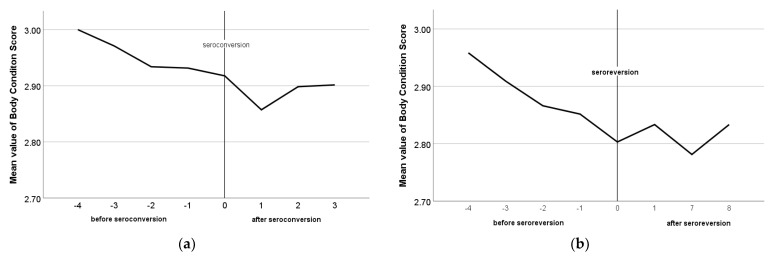
Mean values of body condition score before and after the seroconversion (**a**) and the seroreversion (**b**) incident.

The mean values of BCS varied from 2.8 to 3.0 during the study and the overall mean value of BCS in the studied animals was 2.9 regardless of the serological pattern, breed, and farm. The mean values of BCS in constantly seronegative and seropositive animals are presented in Figure 5. In constantly seronegative animals, BCS reduced until the third sampling occasion, and then increased, whereas, in constantly seropositive animals, BCS reduced until the fourth sampling occasion and increased in the last one. In seroconverted animals, BCS was almost constant at the sampling occasions until the seroconversion incident and decreased at the first sampling occasion after it, whereas, in seroreverted animals, BCS decreased at the sampling occasion of a seroreversion incident and increased in the next one (Figure 6a,b).

During the study, arthritis in at least one limb, respiratory disease, and mastitis were recorded at least once in 23.8% (97/407), 3.4% (14/407), and 3.2% (13/407) of the studied ewes. The frequencies of these health disorders in ewes of different serological patterns are summarized in Table 2. Seroreverted and animals with an intermittent presence of antibodies presented the highest frequency of arthritis, whereas constantly seropositive ewes demonstrated the highest frequency of respiratory disease and mastitis.

### 3.3. Adjusted Relative Risks

Adjusted RRs for serological patterns estimated by binary models are presented in Table 3. A one-year increase in age was associated with an increased RR of an animal being constantly seropositive, by 1.60 times (95% CI, 1.35–1.91, *p* < 0.001). On the other hand, age was negatively associated with the occurrence of the constantly seronegative and the seroconverted patterns; a one-year increase in animal age decreased by 32% (95% CI, 3–67%, *p* < 0.05) and 28% (95% CI, 3–59%, *p* < 0.05) the likelihood of an animal being constantly seronegative or seroconverting, respectively, during the study. Moreover, the intermittent presence of antibodies was 4.53 times (95% CI, 1.61–12.76, *p* < 0.01) more likely to occur in Chios ewes, whereas age was negatively associated with the occurrence of this pattern; a one-year increase was associated with a decreased RR of the intermittent presence of antibodies by 32% (95% CI, 1–72%, *p* < 0.05).

Table 4 summarizes the adjusted RR derived from the repeated measures binary models for (i) seropositivity during the study and (ii) seropositivity in animals with an intermittent presence of antibodies. Age was significantly associated with seropositivity; in particular, the RR for seropositivity during the study increased with age by 1.78 times (95% CI, 1.41–2.25, *p* < 0.001). Also, Lacaune ewes were 2.63 times (95% CI, 1.35–5.00, *p* < 0.01) more likely to be found seropositive during the study. Moreover, the RR of seropositivity was increased by 1.72 times (95% CI, 1.28–2.33, *p* < 0.001) at pre-lambing compared to pre-mating, while seropositivity exclusively in animals with an intermittent presence of antibodies was also increased by 2.78 times (95% CI, 1.48–5.00, *p* < 0.01) during pre-lambing. Also, the year of the study was associated with seropositivity; animals were more likely to be seropositive during the second year of the study compared to the third one (*p* < 0.001), while animals with an intermittent presence of antibodies were more likely to be seropositive during the first or the second year of the study compared to the third one (*p* < 0.05).

Adjusted RRs for the seroconversion and seroreversion incidents are summarized in Table 5. Ewes were 3.23 times (95% CI, 1.85–5.53, *p* < 0.001) more likely to be found seroconverted at pre-lambing compared to pre-mating. Also, the year of the study was associated with seroreversion incident; the RR for seroreversion increased by 20.0 times (95% CI, 3.85–100.00, *p* < 0.001) in the second year of the study and by 33.3 (95% CI, 7.69–100.00, *p* < 0.001) in the last year of the study compared to the first one.

## 4. Discussion

To the best of our knowledge, it is the first time that risk factors of SRLVs infections have been prospectively studied in intensively reared dairy sheep farms in Greece; it is also the first time the effects of these factors on seropositive status and the occurrence of different serological patterns, as well as on seroconversion and seroreversion incidents, have been assessed. Τhe farms enrolled in this study were selected exploiting specific typology and on the basis of being representative of the intensive system applied in dairy sheep farms in Greece; however, the results of the present study cannot be directly generalized and further large-scale studies are warranted for the confirmation of our findings. In any case, our findings are crucial in the investigation of the epidemiology of SRLVs infections in our country and constitute the first evidence of the virus spread and the associated risk factors.

Seroprevalence rates were high in the studied farms (57.5–75.4% during the 2 years of this study), indicating a wide spread of the virus within them. These findings are consistent with two relevant studies conducted in Greece in the past (reporting a seroprevalence equal to 65.0% and 47.0%) [30,31], which however did not include any information regarding the implemented farming systems. The animals in our study were reared under intensive farming systems, which are known to favour SRLVs transmission due to the close contact of animals and the poor ventilation conditions compared to the extensive farming systems [15,18,35,36,37,38,39].

Age has been recognized as a risk factor significantly associated with seropositivity in many studies [4,17,18,37]. This association has been attributed to the late seroconversion of infected animals [40], the establishment of latent infection for a long period after the initial infection of the animals [1], and the increased risk of infection for older animals due to longer exposure to the virus compared to young animals [18]. In our study, it was the first time that age was investigated as a potential risk factor, not only for the seropositive status of individual ewes but also for the manifestation of specific serological patterns and the seroconversion/seroreversion events under a prospective study design. The RR for the seropositive status and the manifestation of the constantly seropositive pattern during the study increased by age ca. 1.8 and 1.6 times, respectively, whereas a constantly seronegative pattern was decreased by age ca. 1.3 times. Moreover, age was negatively associated with the seroconversion incident and the intermittent presence of antibodies during the study. At the beginning of the study, older animals were already seropositive and therefore were classified as constantly seropositive at the end of the study, whereas the infected younger animals seroconverted during the two-year period. The manifestation of an intermittent presence of antibodies in young animals may be associated with a recent infection which has not fully activated the immune system of the animal, while the production of antibodies is unstable and not detectable in some cases.

In our study, the breed was also recognized as a risk factor for animals’ seropositive status during the study. Lacaune ewes were more likely to be found seropositive compared to Chios ewes. Other studies have also suggested a breed effect on the seropositivity, especially for the purebred animals compared to the cross-breed ones [19,22,38]. Lacaune breed is a highly productive dairy sheep breed which has been extensively imported and reared in our country under intensive farming systems over the last two decades. However, even though animal trade has been recognized as a major risk factor for SRLVs transmission, certifications regarding the SRLV status of the farms of origin are not commonly available [18,20]. Therefore, it could be assumed that although breeding stocks trade is not permitted for SRLVs-infected farms, infected animals may be either imported or purchased from local SRLVs-positive farms, which remain undiagnosed due to the inexistent surveillance programs. This means that the precautions for animal importation and animal trade of breeding stocks should be stricter, and the screening of animals should be obligatory and based on efficient diagnostic tests.

Another remarkable finding of this study was the intermittent presence of antibodies, mainly in the Chios ewes. The specific serological pattern could be associated either with a specific immune response of Chios sheep to SRLVs infections or to the low diagnostic effectiveness of the ELISA test applied in our study due to the circulation of more than one viral strain in Chios farms, which could be beyond its diagnostic spectrum. The latter possibility requires not only the molecular investigation of SRLVs infections of the studied animals, but also the identification of the circulating viral strains. The RR of seropositivity in the specific breeds needs to be further investigated in more farms, as in our study, the effect of the breed could be confounded with the effect of the farm, although the random effect of the farm has been considered in the statistical models. Τhe potential genetic susceptibility/resistance to SRLVs infection should be further investigated for specific breeds, and mainly the most productive ones, which are commonly exploited under intensive farming systems.

To our knowledge, it is also the first time that the production stage has been evaluated as a potential risk factor for animal seropositivity. In our study, sampling occasions were predetermined twice during the production cycle, namely, 3–4 weeks before the onset of the mating season and 2–4 weeks before the lambing season. It was found that seropositive status during the study, seropositivity in animals with an intermittent presence of antibodies, and the seroconversion incident were increased at pre-lambing. This could imply an increase of the antibody titer in ewes during the last stage of pregnancy. This finding is not consistent with a previous study where a fall in antibody titer to small ruminant lentivirus was documented in seropositive goats during the last month of pregnancy [41]. Generally, a decline in blood serum IgG antibodies has been observed prepartum both in ewes and cows, explained either by the transfer of IgG antibodies to the udder and the colostrum or the suppressed immunological response of animals during the periparturient period [42,43,44,45]. Also, in a study investigating the periparturient diagnosis of Bovine Viral Diarrhoea virus in cattle, total IgG and IgG1 antibodies were reduced, while IgG2 antibodies increased pre-partum in the studied animals [46]. However, in our case, this cannot explain the increase in antibody titer, as it has been shown that the immunological response of SRLV-infected sheep is restricted in the production of IgG1 antibodies [47]. However, in our study, pre-lambing sampling occasion is likely to coincide with an increase in antibodies production due to the subsequent production of colostrum. Consecutive measurement of anti-SRLVs specific total IgG, IgG1, and IgG2 for a long period pre- and post-partum could elucidate this serological reaction and its association with the lambing period. However, the increased seroconversion incidents at pre-lambing could be explained by previous increased cases of infection during the mating period when ewes come in closer contact with possible infected rams and present intense activity, increasing the risk of horizontal infection. Therefore, it is possible for the ewes infected during the mating period to seroconvert after 4–5 months and be found seropositive at the pre-lambing period. The confirmation of this hypothesis requires the molecular testing of animals at these production stages for the identification of the time-point of infection.

In any case, the increased possibility of an animal being seropositive at pre-lambing should be confirmed and exploited for the designation of control programs. Ιn addition, it should be clarified if the increased possibility of seropositive animals at pre-lambing is associated with increased viral circulation and excretion. It is crucial for the screening programs to be implemented in appropriate production stages to minimize misdiagnosis of animals with antibody fluctuation or recently infected animals. These animals serve as reservoirs of the virus in farms, resulting in the gradual re-emergence of high prevalence rates.

The second and third years of the study were significantly associated with seroreversion incidents compared to the first one. Seroreverted animals in our study belonged mainly to age class 4 (3 < x ≤ 4) at the beginning of the study, and their mean age at the seroreversion incident was 5.2 ± 0.64. These findings indicate a special underlying mechanism of humoral immune response that results in seroreversion for animals that have been seropositive for a long time. Also, the mean age at the seroreversion incident of age class 1 ewes excludes the possibility of seroreversion due to the loss of maternal antibodies, which is observed much earlier. According to the available literature, seroreversion has not been recorded and explored in SRLVs-infected sheep in the past; however, it has been described in human retroviral infections in end-stage HIV patients [48] or in HIV-infected persons treated with antiretroviral therapy [49,50]. The elucidation of the seroreversion mechanism in SRLVs-infected sheep requires the combination of serological and molecular testing for the confirmation of potential viral suppression. Also, the presence of clinical signs associated with SRLVs infections or even with other chronic diseases that may lead to immunological suppression should be investigated in seroreverted animals.

Poor body condition score has been previously reported in seropositive animals [24]. SRLVs cause chronic incurable disease, and progressive weakness and emaciation are observed in some animals with clinical signs [51]. However, none of the studied farms reported a remarkable history of severe clinical cases of SRLVs-compatible disease, and animals were reared under intensive farming conditions, adopting satisfying preventive veterinary protocols (antiparasitic treatments and vaccinations) and appropriate nutrition according to their demands (production stage, productivity, etc.). Moreover, low-yielding ewes were removed from the flock and animals with clinical manifestation of diseases were either treated or readily removed as well. Therefore, it was likely that, despite the high seroprevalence rates, poor body condition score was not observed, as severely SRLVs-infected animals were removed before a remarkable body weight loss was clearly evidenced.

Considering that SRLVs cause chronic infection and disease, the prospective study design to investigate risk factors, not only for animal seropositivity but also for serological patterns, leads to safer conclusions compared to the ones exported by cross-sectional studies. Nonetheless, this study was limited to four intensive dairy sheep farms with high SRLVs’ prevalence rates and investigated specific risk factors at the animal level. The investigation of more risk factors at the animal and farm levels (environmental factors, housing conditions, husbandry measures and management, etc.) in animals reared under various farming systems and for farms with various infection statuses could enrich and extend the findings of this study. In any case, the combination of serological and molecular testing is an essential future objective for the investigation of risk factors for SRLVs infections and the further elucidation of SRLVs epidemiology.

## 5. Conclusions

The present study constitutes the first risk factor analysis for SRLVs infections in Greece. A significant association of pre-lambing period with animal seropositivity and seroconversion incident was evidenced, indicating this period as being more appropriate for serological screening control. Increased age was recognized as a risk factor for the manifestation of the constantly seropositive pattern, whereas the seroconversion, the constantly seronegative pattern, and the intermittent presence of antibodies were more likely to occur in younger animals. Also, the results of our study confirmed the significance of age as a risk factor for SRLVs seropositivity. Seroreversion incidents were increased after a long period of seropositivity, implying the necessity of molecular testing in seronegative old animals for the minimization of false negative serological results. Our findings regarding the association of Lacaune ewes with seropositivity and Chios ewes with the manifestation of an intermittent presence of antibodies underpin the potential breed-related resistance/susceptibility and their possibility to be considered in genetic selection programs. Further and large-scale epidemiological studies, including more animals of various breeds, reared under various farming systems, are warranted for the confirmation of our results and the elucidation of further risk factors aiming at the designation and implementation of successful control programs against SRLVs infections in dairy sheep in Greece.

## Figures and Tables

**Table 1:** Seroprevalence during the study.

Sampling Occasion	Prevalence
First (pre-mating)	57.5% (234/407)
Second (pre-lambing)	70.0% (285/407)
Third (pre-mating)	71.7% (292/407)
Fourth (pre-lambing)	75.4% (307/407)
Fifth (pre-mating)	66.0% (186/282)

**Table 2:** The frequencies of clinical signs associated with SRLV infection in ewes of different serological and infection patterns during the study.

Serological Pattern	Arthritis	Respiratory Disease	Mastitis
Constantly seronegative	14.5% (9/62)	0.0% (0/62)	1.6% (1/62)
Constantly seropositive	21.8% (41/188)	3.7% (7/188)	3.7% (7/188)
Seroconverted	15.9% (13/82)	3.7% (3/82)	4.9% (4/82)
Seroreverted	45.7% (16/35)	5.7% (2/35)	0.0% (0/35)
Intermittent presence of antibodies	45.0% (18/40)	5.0% (2/40)	2.5% (1/40)

**Table 3:** Adjusted relative risks for serological patterns estimated by binary logistic models.

Dependent Variable	Risk Factor	Categories	*β*	Relative Risk	CI_95%_	*p*
Constantly seropositive	Age	-	0.473	1.60	1.35–1.91	<0.001
	Breed *	Chios	0.236	1.27	0.52–3.10	ns
Constantly seronegative	Age	-	−0.272	0.76	0.60–0.97	0.026
	Breed	Chios	−0.253	0.78	0.28–2.19	ns
Seroconverted	Age	-	−0.252	0.78	0.63–0.97	0.022
	Breed	Chios	−0.372	0.69	0.30–1.57	ns
Seroreverted	Age	-	−0.173	0.84	0.64–1.11	ns
	Breed	Chios	0.469	1.60	0.55–4.63	ns
Intermittent presence of antibodies	Age	-	−0.279	0.76	0.58–0.99	0.042
	Breed	Chios	1.510	4.53	1.61–12.76	0.004

CI_95%_: 95% confidence interval; ns: not significant; * Reference category is Lacaune breed.

**Table 4:** Adjusted relative risks for seropositivity during the study and seropositivity in animals with an intermittent presence during the study.

Dependent Variable	Risk Factor	Categories	*β*	Relative Risk	CI_95%_	*p*
Seropositivity during the study	Age	-	0.579	1.78	1.41–2.25	<0.001
	Breed *	Chios	−0.964	0.38	0.20–0.74	0.004
	BCS	-	0.142	1.15	0.46–2.92	ns
	Year of the study *	1 2	0.560 1.032	1.75 2.81	0.81–3.77 1.64–4.80	ns <0.001
	Production stage *	Pre-mating	−0.552	0.58	0.43–0.78	<0.001
Seropositivity in animals with an intermittent presence of antibodies	Age	-	−0.134	0.87	0.70–1.09	ns
	Breed	Chios	−0.101	0.90	0.40–2.02	ns
	BCS	-	−0.630	0.53	0.10–2.88	ns
	Year of the study	1 2	1.383 1.701	3.99 5.48	1.19–13.33 1.35–22.24	0.025 0.018
	Production stage	Pre-mating	−1.011	0.36	0.20–0.68	0.001

CI_95%_: 95% confidence interval; ns: not significant; * Reference categories for breed, year of the study, and production stage were Lacaune breed, third year, and pre-lambing, respectively; BCS: body condition score.

**Table 5:** Adjusted relative risks for seroconversion and seroreversion incident.

Dependent Variable	Risk Factor	Categories	*Β*	Relative Risk	CI_95%_	*p*
Seroconversion incident	Age	-	−0.135	0.87	0.74–1.03	ns
	Breed *	Chios	−0.296	0.74	0.49–1.14	ns
	BCS	-	0.299	1.35	0.48–3.78	ns
	Year of the study *	1 2	0.260 0.082	1.30 1.09	0.47–3.61 0.36–3.3	ns ns
	Production stage *	Pre-mating	−1.162	0.31	0.18–0.54	<0.001
Seroreversion incident	Age	-	−0.077	0.93	0.68–1.26	ns
	Breed	Chios	0.730	2.08	0.82–5.26	ns
	BCS	-	−0.711	0.49	0.03–7.12	ns
	Year of the study	1 2	−3.569 −2.941	0.03 0.05	0.01–0.13 0.01–0.26	<0.001 <0.001
	Production stage	Pre-mating	−0.937	0.39	0.12–1.33	ns

CI_95%_: 95% confidence interval; ns: not significant; * Reference categories for breed, year of the study, and production stage were Lacaune breed, third year, and pre-lambing, respectively; BCS: body condition score.

## Data Availability

The data presented in this study are available on request from the corresponding author. The data are not publicly available due to privacy restrictions.
